# Measurement of Methanol and Ethanol Contents in Most Commonly Used Herbal Distillates Produced by Three Famous Brands

**Published:** 2018-06

**Authors:** Mahdi YOUSEFI, Reza AFSHARI, Masoumeh SADEGHI, Roshanak SALARI

**Affiliations:** 1. Dept. of Persian Medicine, School of Traditional Medicine, Mashhad University of Medical Sciences, Mashhad, Iran; 2. Medical Toxicology Research Center, Mashhad University of Medical Sciences, Mashhad, Iran; 3. Dept. of Epidemiology, School of Public Health, Tehran University of Medical Sciences, Tehran, Iran; 4. Dept. of Pharmaceutical Sciences, School of Traditional Medicine, Mashhad University of Medical Sciences, Mashhad, Iran

**Keywords:** Herbal distillate, Herbal water, Traditional Persian medicine, Methanol, Ethanol

## Abstract

**Background:**

Herbal distillates have been used for many centuries as herbal medicines in Traditional Persian medicine. The main purpose of this study was to determine methanol and ethanol contents in commonly-used industrial herbal distillates produced by three famous factories in Khorasan Razavi, Mashhad, Iran (2014–2015).

**Methods:**

Ninety herbal waters of ten types of most commonly used herbal distillates including Willow *(Salix alba)*, Ajava seeds (*Carum copticum*), Fennel (*Foeniculum vulgare***)**, Poleigamander *(Teucrium polium)*, Forty plants, Peppermint *(Mentha piperita)*, Camel thorn *(Alhagi camelorum)*, Chicory *(Cichorium intybus)*, Fumitory *(Fumaria officinalis)* and Rose water *(Rosa damascene)* of three famous company in Mashhad were randomly bought from market. Methanol and ethanol contents of each sample were measured by Gas chromatography according to the standard method. Collected data were analyzed by SPSS using appropriate descriptive statistical tests.

**Results:**

The highest average amount of methanol of all tested distillates is for forty plants (46.06 mg/dl) and *M. piperita* (46.72 mg/dl) and the lowest for ajava seed (8.46 mg/dl). The maximum and minimum ethanol level was reported for rose water (0.39 mg/dl) and ajava seed (0.15 mg/dl), respectively.

**Conclusion:**

High methanol and ethanol concentrations may induce toxicity in people taking these products regularly for a long time. Therefore, considering the harmful effects of methanol and ethanol on human health, further studies are required for determining permitted levels of methanol and ethanol in herbal distillates.

## Introduction

The first clear evidence of distillation comes from Greek chemists working in Alexandria in the first century AD (1). The Chinese may have independently developed the practice around the same time (2). Distilled water was described in the second century AD by Alexander of Aphrodisias (3). Muslim scientists such as Muhammad ibn Zakariyā Rāzi and Abu Mūsā Jābir ibn Hayyān stated about distillation and its instrument called “taghtir” and “ghare- va- anbiq” in Persian medicine, respectively. Muhammad ibn Zakariyā Rāzī (Latinized name Rhazes or Rasis), was the discoverer of alcohol. Herbal water (plant water, herbal essence, aromatic water) is one of the common forms of medications in traditional Iranian medicine especially in recent 200 years (4). Nowadays, frequent production and prescription of herbal distillates make the measurement of methanol and ethanol contents in these products, necessary.

Herbal water is referred to the liquid obtained from the cooled steam (distillation) of medicinal plants. It can also be called aqueous herbal extract which is a water-based preparation of a plant containing the biological active portion of the plant without its residue. Herbal waters are produced from various parts of plants and alcohols such as methanol and ethanol are the products of plant’s fibers fermentation (5). Methanol is the simplest type of alcohol that its oral use and even vapor exposure is extremely toxic for humans (6). Methanol, also named methyl alcohol, wood spirit, carbinol, wood alcohol, or wood naphtha, is a colorless and volatile liquid with a characteristic odor, primarily used for manufacturing other chemicals and as a solvent, glass cleaner, windshield washer fluid, carburetor cleaner, de-icing solution, paint remover, varnish, photocopying fluid, canned solid (picnic) fuel, and small engine fuel. Methanol may also be found as an adulterant of alcoholic drinks. Methanol is highly toxic following ingestion; inhalation or dermal exposure. Ingestion is the most common route of exposure. Susceptibility to methanol poisoning varies greatly and ingestion of 0.25 mL/kg of 100% methanol (pure methanol) would theoretically, assuming complete absorption, results in severe toxicity. Death has also been reported after ingestion of about 15 mL of 40% methanol (7, 8).

Methanol’s toxic effects usually manifest a few hours after consumption. Methanol causes different gastrointestinal disorders such as pain, nausea and vomiting. Most patients note visual disturbances as one of the first symptoms. Visual impairment, secondary to optic nerve necrosis or demyelination, ranging from blurred vision to visual field deficits and even total blindness may be seen. Severe metabolic acidosis, hypotension, central nervous system depression, confusion, and ataxia are other common observable signs. Methanol in high doses can induce coma and in some cases, death (7, 8). The majority of the existing information about methanol toxicity is related to acute rather than chronic exposure. The toxic effects of repeated or prolonged exposures to methanol are supposed to be similar but less severe than those induced by acute exposure. High solubility of methanol in oil and water is the main reason for its toxicity. At first, methanol is dissolved in total body water. Then it accumulates in cerebrospinal fluid and reaches higher concentrations of acceptable ranges (9). Toxicity of methanol is due to its oxidation by alcohol dehydrogenase and aldehyde dehydrogenase and producing formic acid which is the metabolite of methanol (9–11). These effects may include gastrointestinal (abdominal pain, nausea, and vomiting), ophthalmic (irritation, blurred vision), central nervous system (headaches, unsteadiness, dizziness, tinnitus, hearing loss, seizures, amnesia, anxiety, phobias) and other nonspecific symptoms (weakness, malaise, fatigue, palpitation). This wide range of symptoms may falsely mimic some chronic diseases such as fibromyalgia, myasthenia gravis (MS), systemic lupus erythematous (SLE), diabetes mellitus, chronic fatigue syndrome, Alzheimer, and attention disorder (12). For example, in a case report, metabolic acidosis due to methanol poisoning that causes many complications such as cerebral edema, have been reported (13). Hence diagnosis of chronic methanol toxicity is highly important, although low-level exposure of methanol may be asymptomatic. Frequent multiple exposures; particularly oral intake may induce toxicity. The acceptable blood level of methanol is less than 20 mg/dl (9–11, 14, 15).

When the level of ethanol in the blood is greater than 50–100 mg/dl, symptoms of ethanol poisoning such as hypoglycemia, coma and hyperthermia occur. The possibility of these complications in people with lower glycogen stores is also possible at lower doses of ethanol. Although ethanol level in distillates and its toxicity is less than methanol, its determination is necessary because of its frequent use especially in children (16).

Various factors, such as plant type, fermentation temperature, etc., are involved in the formation of methanol and ethanol in herbal waters. Since these products are used daily by people at home, their safety and the amount of toxic free compounds like methanol and ethanol are very vital to consumers.

Therefore this study was conducted to determine methanol and ethanol contents in commonly-used industrial herbal distillates produced by three famous factories of Mashhad in 2014–2015.

## Materials and Methods

Ten types of the most commonly used herbal distillates including peppermint (*Mentha piperita*), willow (*Salix alba*), rose water (*Rosa damascene*), camelthorn)*Alhagi camelorum*), ajava seeds (*Carum copticum*), fennel (*Foeniculum vulgare*), fumitory (*Fumaria officinalis*), chicory (*Cichorium intybus*), poleigamander) *Teucrium polium* (and forty plants were purchased from three main factories (A, B and C) in Mashhad, Iran. Three samples of each herbal water with different production dates (new= about 2 months, middle = between 2–7 months and old samples= more than 7 months after the production) were bought from each factory. Thus, a total of 90 bottles were purchased and transferred to the laboratory for determination of methanol and ethanol concentrations. Forty plants distillate usually used for gastrointestinal upsets is one of the frequently used compound distillates. This distillate is not made based on the original traditional medicine literature known as a folklore formulation. Ingredients of this distillate are different from one formulation to another. According to manufacturers the composition of forty plants distillate is mostly constant in each factory but not the same as other factories. The formulation of this distillate was patent for studied company and not opened for investigators.

The production and expiration date of all samples were controlled and recorded. All samples have not been expired. All 90 samples were collected in original containers and were not opened before sampling in laboratory. Regarding the ethical aspects of research, manufacturers were defined with codes and the examiners did not know anything about the producers.

Gas chromatography (GC) was used for analyzing the concentrations of methanol and ethanol in each company products. The brand of GC device was Varian CP-3800 chromatograph equipped with a capillary column. The characteristics of column coated with silica CP-sil5CB were (length: 30 m, inside diameter: 0.25 mm, outside diameter: 0.39 mm, film thickness: 0.10 μm) with flame ionization detector (FID). The oven was programmed from 40 to 210 °C at a rate of 20 °C/min. Injection port and FID temperatures were 170 °C and 280 °C, respectively. The neutral carrier gas was hydrogen with a flow rate of 30 ml/min. Each sample was measured three times and the mean value was the reported value. Accuracy, precision, and reproducibility of the method were 97%, 96% and 98%, respectively.

Data analysis was carried out by SPSS software (Ver. 19, Chicago, IL, USA).

## Results

The average methanol contents of distillates produced by different companies at 3-time intervals of production are shown in Table 1. The average amount of methanol of a distillate was also calculated in different companies where the maximum amount was respectively as follows:

**Table 1:** Methanol and Ethanol concentrations in studied distillates for each industrial unit (Factory)

***Type of herbal distillate***	***Time of production (month)***	***Methanol concentration (mg/dl) Factory***			***Ethanol concentration (mg/dl) Factory***		
		***A***	***B***	***C***	***A***	***B***	***C***
Chicory	< 2	10.90	13.81	22.51	0.14	0.10	0.05
	2–7	13.21	26.65	17.93	0.04	0.03	0.06
	> 7	13.90	10.95	17.11	1.40	0.17	0.43
Fumitory	< 2	10.09	26.89	12.46	0.19	0.18	0.12
	2–7	9.13	12.57	22.53	0.03	0.07	0.16
	> 7	28.21	21.15	15.50	0.89	0.53	0.40
Camelthorn	< 2	19.77	19.28	115.33	1.36	0.15	1.07
	2–7	11.59	14.82	40.62	0.07	0.08	0.36
	> 7	38.30	10.76	50.93	1.54	0.55	0.37
Rose water	< 2	5.36	29.55	8.52	13.68	142.53	29.01
	2–7	9.64	12.36	6.86	53.65	17.26	13.62
	> 7	12.13	12.44	6.69	49.56	10.51	21.15
Forty plants	< 2	8.76	192.10	19.18	0.05	0.12	1.07
	2–7	12.32	51.95	21.95	0.23	0.94	0.44
	> 7	31.85	34.93	41.50	0.69	1.65	0.68
Ajava seeds	< 2	8.71	11.12	4.26	0.03	0.06	0.39
	2–7	7.95	16.15	4.50	0.00	0.05	0.00
	> 7	12.65	7.57	3.25	0.65	0.01	0.13
Poleigamander	< 2	11.15	40.17	28.51	0.44	0.83	2.58
	2–7	6.65	37.33	21.21	0.85	0.02	1.83
	> 7	17.45	19.41	10.25	2.32	0.45	0.71
Willow	< 2	7.03	24.92	13.41	0.00	0.32	0.83
	2–7	9.74	7.77	15.77	1.21	0.64	0.58
	> 7	8.87	10.91	11.51	0.11	0.28	0.43
*Mentha piperita*	< 2	19.54	177.28	40.06	0.32	2.32	0.87
	2–7	19.55	26.68	24.36	1.56	0.20	0.84
	> 7	13.12	77.65	22.22	0.90	3.82	0.17
Fennel	< 2	15.27	9.25	2.53	0.32	0.04	0.64
	2–7	23.11	9.42	2.80	0.86	0.02	0.25
	> 7	12.41	7.70	2.65	0.62	0.21	0.09

*M. piperita* distillate produced by Company B (mean value: 93.87 mg/dl), forty plant distillate of the same company (92.99 mg/dl) and the lowest rate was for fennel (2.66 mg/dl) produced by Company C. Average methanol level in produced distillates (10 studied distillates) was 14.28 mg/dl, 32.45 mg/dl and 20.90 mg/dl for A, B and C Companies, respectively. The highest average amount of methanol of all tested distillates is for forty plants (46.06 mg/dl) and *M. piperita* (46.72 mg/dl) and the lowest for ajava seed (8.46 mg/dl). The same calculations were also performed on the ethanol content of distillates, which represents the highest amount for rose water of company B (56.77 mg/dl) and then rose water produced by company A (38.97 mg/dl). The lowest amount was for ajava seed distillate (0.04 mg/dl) produced by company B.

The average ethanol content of distillates produced by A, B and C companies was 4.46 mg/dl, 6.14 mg/dl, and 2.64 mg/dl, respectively. The maximum and minimum ethanol level in all the tested distillates was reported for rose water (0.39 mg/dl) and ajava seed (0.15 mg/dl), respectively. The index of the methanol and ethanol level in different distillates shows some changes over time in such a way that the amount of methanol in company A increases in all distillates (except *M. piperita* and fennel distillates) and the amount of ethanol in all distillates of this company usually increases, by an increase in distillates shelf life. But the amount of methanol in all distillates produced by company B was reduced over time and the amount of ethanol was increased for all distillates with the exception of rose water, ajava seed, and poleigamander distillates. In most distillates produced by company C, the amounts of methanol and ethanol were reduced over time. Average amount of methanol for distillates of each company was analyzed separately, in which a significant difference was not seen in all cases except the forty plants distillate produced by company C (*P*=0.001). The amount of ethanol was not significant for all distillates except for forty plants distillate produced by C and A companies (*P*<0.001). The average amounts of methanol and ethanol in all tested distillates were compared in 3 companies, and the results showed no significant differences in this regard (Table 2).

**Table 2:** Comparison of overall Methanol and Ethanol concentrations of the companies under study (results of Kruskal-Wallis Test)

***Factory***	***Methanol***			***Ethanol***		
	***Mean Rank***	***X^2^***	***P-Value***	***Mean Rank***	***X^2^***	***P-Value***
A	38.28	4.82	0.09	47.73	2.01	0.37
B	53.07			40.00		
C	45.17			48.77		

## Discussion

History of using herbal distillates for therapeutic purposes has long been practiced in many countries. Herbal distillates can be extracted using traditional and industrial methods. Consumption rate of distillates and their production rate are increasing in companies in a parallel fashion. In both traditional and industrial processes, methanol will be produced due to the keeping of the wood-bearing plant in the water. The duration of keeping of the plant in the water before heating and the commencement of the distilling process will have an impact on the augmentation amount of methanol and ethanol. Other important factors in the production of methanol and ethanol include wooden parts of the plant, ambient temperature, uncapped bottles containing distillates, soaking time, plant varieties and the material of collection or storage containers of the distillates and even distillates pasteurization (17–20). Alcohol level in distillates been changed by passing time. Distillates are not totally free of carbohydrates or other alcohol convertible components. Some chemical reactions happen during the storage and maintenance of these products and changes such as discoloring, disodoring, bittering or sedimentation are not infrequent in lasted distillates especially in inappropriate conditions including sunshine or high-temperature exposure. In order to evaluate the effect of time passing on methanol and ethanol contents of distillates, three samples with different production dates have been gathered for analysis (21, 22). Concentration of the methanol in herbal distillates produced was compared using traditional and industrial methods. Three samples of each widely used herbal distillates in Arak city including musk willow, *M. piperita*, fenugreek, camelthorn, dill and chicory prepared using traditional methods were compared with the same type of distillates but produced by companies in terms of the methanol concentration. The results showed that there is no significant difference between the concentrations of methanol in these distillates. The highest concentration of methanol in handmade products was obtained for musk willow distillate (266.02 ppm) and the lowest in one fenugreek sample (26.60 ppm). In industrial products, the highest and the lowest concentrations of methanol were obtained in a *M. piperita* sample (415.04 ppm) and musk willow sample (88.08 ppm). The average amount of methanol in the handmade distillates was less than the same samples produced by the industrial method (9).

The methanol amount of 6 widely used herbal distillates was measured in Urmia. They tested dill, *M. piperita*, musk willow, lemon balm, rose water and oregano and stated that the amount of methanol was significantly less and higher in rose water and musk willow distillates, respectively compared with other distillates (23).

The highest and lowest amounts of methanol were reported in dill distillate (*A. graveolens*) (147707±23.8 ppm) and musk willow (79.4±3 ppm), respectively (24). A blindness case was reported following daily consumption of 200 ml of forty plants distillate for six months. The highest and the lowest methanol contents were obtained for dill distillate) 1208± 202.74 mg / l) and ajava seeds distillate (18.93± 1.04 mg / l) (5). In most cases, the amount of methanol is higher than the usual dosage range so that usual consumption of distillates can cause different complications. To reduce methanol concentration, it is needed to improve various steps of processing herbs. Since methanol and ethanol limits are not specified in herbal extracts, the authorities impose certain rules to control these products better than previous. In most distillates, ethanol concentration is in a tolerable range but not free of this alcohol.

The minimum and maximum ethanol and methanol concentrations were different for each company and no same pattern was seen. This difference may be due to the following reasons:

1. The distillates were tested at different intervals of production time; the used plant species might be different at each production stage.
2. With regard to the dispersion of data and lack of their normal distribution, the production process is the same, plant samples used by various companies, were different.
3. Samples randomly collected from different parts of Mashhad City using stratified sampling. The distillates storage conditions were somewhat different in different places.
4. Due to the lack of some plants in some seasons, the possibility of staleness or unfavorable keeping conditions in some plants is not farfetched.
5. Due to the low volume of flowers or fruits, the wooden parts of the plant may be used in the distilling process and consequently methanol level may be high if the wooden parts (like stems, branches, and roots) are used more in this process.
6. It is possible in some cases to use ready essences for distillates preparation as a way to reduce the production costs.

## Conclusion

It is essential to measure the methanol and ethanol contents of distillates in certain time periods due to the complications come from methanol and ethanol toxicities. There is very large dispersion in the methanol and ethanol contents of distillates thus, careful monitoring and precision are needed. Further studies are needed to investigate the effects of different variables such as raw materials, shelf life, production process, storage procedure, as well as large number of samples of methanol and ethanol contents.

## Ethical considerations

Ethical issues (Including plagiarism, informed consent, misconduct, data fabrication and/or falsification, double publication and/or submission, redundancy, etc.) have been completely observed by the authors.
